# The Potential of Deep Roots to Mitigate Impacts of Heatwaves and Declining Rainfall on Pastures in Southeast Australia

**DOI:** 10.3390/plants10081641

**Published:** 2021-08-10

**Authors:** Rachelle Meyer, Alexandria Sinnett, Ruchika Perera, Brendan Cullen, Bill Malcolm, Richard J. Eckard

**Affiliations:** Faculty of Veterinary and Agricultural Sciences, University of Melbourne, Parkville, VIC 3010, Australia; a.sinnett@unimelb.edu.au (A.S.); pererat@student.unimelb.edu.au (R.P.); bcullen@unimelb.edu.au (B.C.); b.malcolm@unimelb.edu.au (B.M.); rjeckard@unimelb.edu.au (R.J.E.)

**Keywords:** heat stress, drought, climate impacts, pasture systems, adaptation

## Abstract

Declines in growing-season rainfall and increases in the frequency of heatwaves in southern Australia necessitate effective adaptation. The Sustainable Grazing Systems Pasture Model (SGS) was used to model the growth of three pasture species differing in root depth and root distribution under three different climate scenarios at two sites. The modelled metabolisable energy intake (in MJ) was used in a partial discounted net cash flow budget. Both the biophysical and economic modelling suggest that deep roots were advantageous in all climate scenarios at the long growing season site but provided no to little advantage at the short growing season site, likely due to the deep-rooted species drying out the soil profile earlier. In scenarios including climate change, the DM production of the deep-rooted species at the long growing season site averaged 386 kg/ha/year more than the more shallow-rooted species, while at the site with a shorter growing season it averaged 205 kg/ha/year less than the shallower-rooted species. The timing of the extra growth and pasture persistence strongly influenced the extent of the benefit. At the short growing season site other adaptation options such as summer dormancy will likely be necessary.

## 1. Introduction

Heatwaves and droughts affecting Australian pastures are becoming more frequent. Rainfall in southern Australia from 1996 to 2015 declined 11% compared to the long-term average beginning in 1900. It is projected that years with below average growing-season rainfall will become more frequent than in the past [1]. Changes in summer rainfall are less certain. In southern Australia, the expected time of drought is projected to increase [2]. Average temperatures in Australia have increased by about 1 °C since 1990. As average temperatures increase the frequency of extreme heat events also increase. High temperatures, defined as two standard deviations above the 1951 to 1980 mean, occurred 2.2% of the time during that period. The frequency of these high temperatures increased to 11.45% during the period from 2001 to 2015 [1]. Under an 8.5 representative concentration pathway (RCP), which assumes continued increases in greenhouse gas emissions, the number of ‘warm spell’ days (6 or more days above the 90th percentile value for daily temperatures from 1961 to 1990) is projected to increase by 100 days per year by 2090 [3].

Livestock producers may adapt by switching to deeper-rooted pasture species which have better growth and persistence under drought and heat-stress conditions [4,5]. Pastures in the high-rainfall zone are typically sown with perennial ryegrass (*Lolium perenne*) and subterranean (*Trifolium subterraneum*) or white clover (*T. repens*). A limitation of more shallow-rooted species like perennial ryegrass is that it is sensitive to heat stress and drought [4,6]. The more deep-rooted, tall fescue has been shown to outperform perennial ryegrass both in growth and physiological functions under abiotic stresses including heat stress and drought due to its deep rooting nature [5]. In addition, Jiang and Huang [5] reported that cell membrane damage and photosynthesis rate reduction was less in tall fescue compared to perennial ryegrass at the end of 21 days of 35/30 °C, day/night and drought. In a study performed in Wales, tall fescue persisted longer than perennial ryegrass under water stress [7].

Deep roots are considered a drought avoidance mechanism, a type of drought resistance in plants [8]. Deeper-rooted plants maintain high tissue water potential by taking up water from the deep soil layers so that they can grow more into the drought period [9]. Since the foliage growth of pastures is related to the supply of water to the growing buds [10], a continuous uptake of soil moisture is important for the growth of pasture into the dry periods. Greater water uptake leads to higher leaf water content, photosynthetic activity, reduced stomatal resistance, and transpiration cooling [5].

Quantifying the extent to which pastures comprised of deeper-rooted species will perform better and provide a resilient feed base in response to heatwaves; in conjunction with projected changes in rainfall, is necessary to determine the potential economic benefit of this management option. Previous modelling has shown that adaptations such as heat tolerance and responsiveness to CO_2_ concentrations outperformed deep roots at a cool and wet site in Tasmania. However, the traits that provided the most advantage varied with the site [11].

The aim of this research was to estimate the benefits of replacing a shallower-rooted species with an intermediate or a deeper-rooted species to ameliorate the impacts of heat stress and projected declines in rainfall on pastures. The analysis expands on previous modelling by incorporating a climate scenario with increased variability and an economic analysis that addresses the role of increased persistence that is often associated with deeper roots. The metabolizable energy (ME) intake and nitrogen use information from the biophysical modelling was used to inform the economic analysis.

## 2. Results

### 2.1. Biophysical Results

#### 2.1.1. Climate Projections

Characteristics of the climate scenarios generated are shown in Table 1. The average of temperature and rainfall of the climate scenarios at the two sites during the growing (April to October) and non-growing seasons (November–March) are displayed. The Future and Extremes scenarios had identical rainfall and average maximum temperatures (Table 1). However, the variability in temperature was greater in the Extremes scenarios than the Future scenarios. The methods of calculating projected minimum temperature in the Future and Extremes scenarios were different, resulting in lower average minimum temperatures in scenarios with increased variability (Extremes). Any differences between the growth of the pasture species between Future and Extremes scenarios reflects only differences in the projected temperature.

Climate change substantially increased the days of heat stress at both sites, both days with maximum temperature 35 °C or above and days with average temperature 23 °C or above (Table 2). The Extremes scenario had more heat stress days from March to October than the Future scenarios at both sites. The increase was due to greater frequency of high temperatures (≥35 °C) under the Extremes scenarios at both sites. In contrast, the lower minimum temperatures in the Extremes scenarios led to slightly fewer heat stress days due to higher average temperature (≥23 °C). This results in slightly lower number of heat stress days in summer, particularly at the short-growing season site (Table 2).

#### 2.1.2. Growth

**Figure 1:**
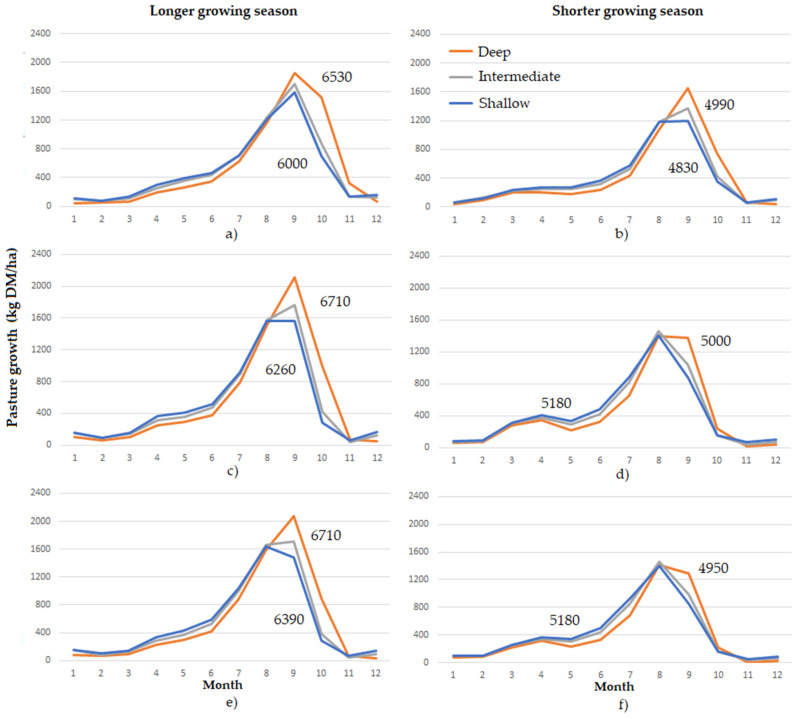
Monthly average growth of three grass species with differing root distributions at two sites and three climate scenarios. The values displayed are annual pasture growth (kg DM/ha/year) for the deep and shallow rooted species. (**a**) Longer growing-season site, historic climate (**b**) Shorter growing-season site, historic climate (**c**) Longer growing season, Future (**d**) Shorter growing season, Future (**e**) Longer growing season, Extremes (**f**) Shorter growing season, Extremes.

The average monthly growth for all scenarios is displayed in Figure 1. Increases in winter temperatures and higher CO_2_ concentrations associated with climate change increased annual DM production under both the Future and Extremes climate scenarios at both the long and short growing season sites. However, spring growth and intake of all pasture species was reduced under Future and Extremes scenarios at both sites (Table 3).

The advantage of deep roots was apparent in spring at both sites. At the longer growing season site this led to the deep-rooted species having the greatest annual DM production and the intermediate species outperforming the shallow rooting species. However, this benefit of deep roots in spring was marginal at the short growing season site in the Future and Extreme scenarios. Further, at the short growing season site annual DM production was lower and more variable for the deeper-rooted pasture species compared with the shallower-rooted species (Table 3). The intermediate root distribution resulted in growth that was more like the shallower rooted species than the deeper-rooted species across scenarios. Root growth followed the same trends as above ground growth (results not shown).

Trends in the intake of the pasture ME were similar to dry matter production at the long growing season site, although the benefit continued into the summer. At the shorter-growing season site, the variability in annual intake of the shallow-rooted species was greater than the deep-rooted species. However, the annual average intake of the deep-rooted species at the short growing season site was greater than the shallow-rooted species in Future scenarios, in contrast to dry matter production (Table 3).

#### 2.1.3. Soil Moisture

**Figure 2:**
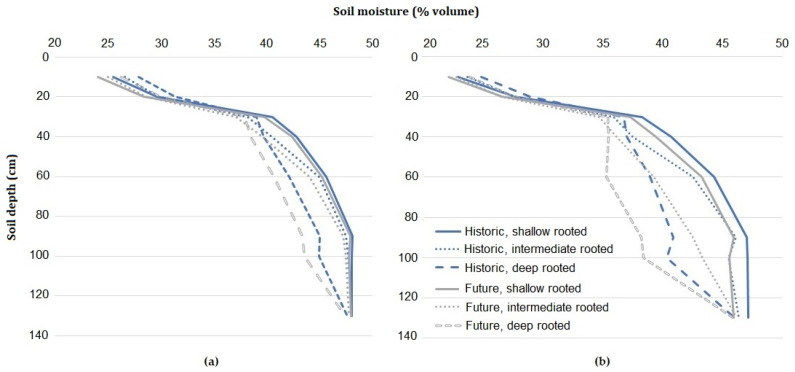
The average soil moisture (% volumetric) at varying depths during spring (September-November) for 12 scenarios (**a**) scenarios at the longer growing season site (**b**) scenarios at the shorter growing season site. Blue lines are historic scenarios and grey lines are Future scenarios.

In the spring, the deeper-rooted species reduced soil moisture at depths between 30 to 130 cm compared to the shallow-rooted species across sites and climate scenarios (Figure 2). The reduction was more pronounced on the shorter-growing season site, with soil moisture differences between 60 and 100 cm depth between 5.3% and 7.9% lower in the historic and Future scenarios. At the longer-growing season site this difference ranged from 3.1% to 4.5%. The intermediate root distribution resulted in soil moisture values midway between the deep and shallow species at the shorter growing site and slightly more similar to the shallow-rooted species on the longer growing season site.

### 2.2. Economic Results

#### 2.2.1. Longer-Growing Season Site

**Figure 3:**
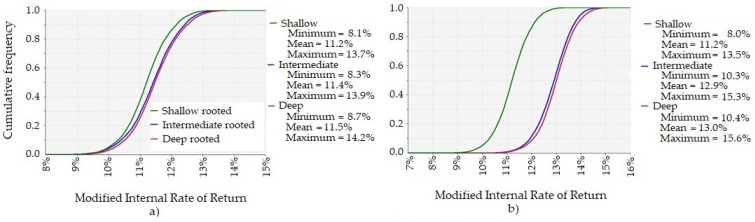
Cumulative Distribution Function of the Modified Internal Rate of Return (MIRR). The real return on the extra capital invested, per annum, for investing in pasture sown with a pasture species that is the same in every way except in the depth of the roots and persistence, under the Extremes scenario in the high-rainfall area over 10,000 simulations (**a**) MIRR if the persistence of the pasture is same for each pasture species (**b**) MIRR if the intermediate and deep root pasture species persisted for 15 years and the shallow rooted pasture species persisted for ten years.

**Figure 4:**
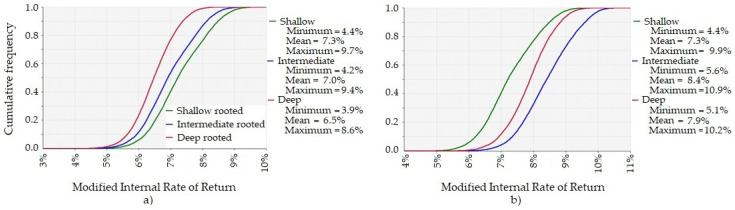
Cumulative Distribution Function of the Modified Internal Rate of Return (MIRR). The real return on the extra capital invested, per annum, for an investment in pasture improvement sown with a pasture species that is the same in every way except in the depth of the roots and persistence, under the Extremes scenario in the shorter-growing season region. (**a**) MIRR if the persistence of the pasture is same for each pasture species. (**b**) MIRR if the intermediate and deep root pasture species persisted for 15 years, and the shallow rooted pasture species persisted for ten years.

The attractiveness of an investment in improved pasture growth depends on the opportunity cost of the capital and the farmer’s attitude to risk. On the longer-growing season site, improving a 100 ha paddock with any of the proposed pasture species has a 70% chance of earning at least an average of 11% real return on capital over the life of the investment. A typical opportunity cost of marginal capital used for pasture, and which could be invested elsewhere on the farm with similar risk could be between 8% and 15% real return. The probability of earning a good return on marginal capital invested in improved pasture is greater if a pasture species with deeper roots than a shallow root species is sown. That is, for every level of return there is a greater probability of earning the return better than the opportunity cost of capital if a pasture species with deep roots is sown. The extra value from sowing a pasture species with deeper roots depends on the value of pasture through the production year as well as the persistence of such a pasture species (see Figure 3). The results in Figure 3 and Figure 4 are based on the pasture being highly valued in January to August and of less value in September to December. If the pasture is highly valued in October to June and of less value in July to September (results not shown)—the curves of the cumulative distribution function shifts to the right and increases the likelihood of earning a higher return from the investment on both sites.

#### 2.2.2. Shorter-Growing Season Site

On the shorter growing season site, improving a 100 ha paddock with any of the proposed pasture species has a 70% chance of earning at least an average of 7% real return on capital over the life of the investment. If the pasture species have the same establishment and persistence characteristics, the modelled deeper-rooted pasture species at the shorter growing season site has no probability of producing more net benefits than the traditional shallow-rooted species for a farm business, under any climate scenario. That is, for every level of return there is a greater probability of earning the return on capital that exceeds opportunity cost, and by a greater margin, if a pasture species with shallow roots is sown. Even if pasture is more valuable during October to June, a deep-rooted pasture species is likely to earn lower returns compared to a shallow-rooted pasture species in a region with low rainfall. If the pasture species have the same establishment but different persistence characteristics, then sowing a pasture species with roots that are intermediate depth or deeper roots, are likely to produce more net benefits than sowing a shallow rooted pasture species with reduced persistence characteristics (see Figure 4).

## 3. Discussion

### 3.1. Biophysical Performance of Pasture Species

Improving pastures with a species with increased rooting depth may not necessarily be adaptive. The benefits of a deeper-rooted pasture species differed for the two case study locations, with the advantages more evident on the long growing season site.

The large difference in the benefit of deeper roots between the two locations is likely due to water availability. During the transition period from late spring to early summer when daily temperatures can cause heat stress and residual soil moisture is still available, the deeper-rooted species can access residual soil moisture. If residual soil moisture is insufficient, the deeper-rooted species does not have an advantage over a shallow rooted species in hot conditions. In an environment where water is limited, the results suggest that deeper roots are not an effective trait to help with adapting to heat stress, as this ability depends more on water availability. Additionally, deeper-rooted species may exacerbate water stress at the end of the growing season through drying down the soil profile earlier. Experimental evidence shows that a deeper-rooted species such as tall fescue extract available soil moisture faster (potentially resulting in a lower relative soil moisture content) and increases evapotranspiration rates compared to more shallow rooted species, such as perennial ryegrass [5]. This would accelerate the utilisation of available water exacerbating soil moisture stress in late spring at the low rainfall site.

Similar limitations of deeper-rooted species have been reported previously. In a study of the performance of tall fescue cultivars, summer active cultivars survived better during spring-summer drought at two high-rainfall sites in Victoria (with average annual rainfall of 477 mm and 596 mm and spring-summer moisture deficits of 533 mm and 460 mm respectively) than at sites in New South Wales with similar rainfall but with a spring-summer water deficit of 650 mm [12]. A modelling study also demonstrated that site characteristics influenced the advantage provided by deep roots [11].

### 3.2. Climate Change Impacts on Pasture Growth

This research has suggested that under the hotter and drier conditions of climate change there will be a change in the time of the year when pasture is grown. Compared to the historic scenario, under climate change scenarios spring pasture growth was reduced and winter growth increased for all pasture species (Table 3). This is consistent with current pasture growth in southeast Australia being limited by low winter temperatures [13]. It suggests that the previously observed declines in spring growth and increases in winter growth [14] will continue.

The change in timing of pasture production with increasing production in winter and reduced production in spring is especially relevant to the economic analysis. The value of a deeper-rooted pasture species will depend on whether the extra dry matter grown is at a time when extra pasture supply would have been limited and in deficit to animal demand and the volatility surrounding the amount of extra pasture grown. If spring becomes a time of year when pasture is in deficit to animal demand, extra spring growth becomes more valuable to a grazier. In this situation, the deeper-rooted species is more advantageous because the deeper-rooted species is expected to grow more pasture in spring compared with a shallower-rooted species. There are other consequences of this change in timing, since key strategic decisions like calving/lambing time are usually timed to coincide with the highest producing months to meet the animal feed demand [15].

### 3.3. Study Limitations

To isolate the productivity differences of a deeper rooting characteristic other aspects of the systems in the two areas were kept constant. Although this assists in quantifying the benefits of deep roots, it leads to modelled systems that are generic. The model does not explicitly incorporate ploidy, time of flowering or endophytes. To eliminate the presence of confounding factors more realistic details were excluded. Scenarios do not include management differences that would likely occur in farms of these areas; legumes that would often co-occur in pastures are not included; and nitrogen limitation that is common, particularly in spring, was avoided. These factors, in addition to the differences that would occur in lighter or heavier soils should be considered when considering if these results are applicable to specific sites in Victoria.

### 3.4. Importance of Pasture Persistence

The importance of pasture persistence to pasture improvement decisions has been highlighted in this research. In the scenario in which the deeper-rooted pasture species persisted longer than the shallower-rooted species it had a greater chance of returning a higher return to marginal capital on both the long and short growing season sites. This finding is supported by Malcolm et al. [16] who found that the longer a pasture persists at peak level of annual production the more profitable it is. Given its importance, the lack of incorporation of pasture mortality and persistence in many biophysical models suggests this should be addressed when using such models to determine impacts and alternative adaptation options. This analysis addresses this gap with the economic analysis, but it is often overlooked.

The results of the current study suggest that researchers looking for a pasture adaptation response to a changing climate, in dry areas, should consider characteristics other than increased root depth alone, including increased persistence and summer dormancy. Mediterranean tall fescue, which is summer dormant, has been shown to have good persistence in areas with dry summers [17,18]. In New South Wales, Mediterranean tall fescue was shown to experience high mortality during a drought but gradually recovered [12]. Summer active tall fescue is suitable in areas where annual rainfall >600 mm with some amount of summer rainfall or in heavy textured clay soils that hold some residual moisture in the subsoil layers in latter part of the growing season [19]. Phalaris could be considered instead of summer active tall fescue for areas prone to drought and heat because of the summer dormancy and superior heat and drought tolerance characteristics [20,21]. In a biophysical modelling study, Ghahramani and Moore [22] found adding summer active lucerne, phosphorous fertilizer and confinement feeding were the best options to cope with increasing temperatures and decreasing rainfall associated with climate change. However, that study did not consider climate extremes.

## 4. Materials and Methods

### 4.1. Biophysical Modelling

This study used whole-farm system modelling to estimate differences in annual production from a modelled pasture with three root architectures: shallow, intermediate, and deep. The dynamic whole-farm system model SGS was used [23]. SGS incorporates soil water, soil nutrients, pasture, and animals. These components are influenced by climate and management actions as well as interactions between them. For instance, dung and urine from livestock influence soil nutrients and pasture growth and digestibility, which will influence pasture intake. The SGS model’s pasture and water routines have been validated for the regions of interest [24,25,26] and has been used in similar studies [11,27]. A limitation of the SGS model is that it does not predict pasture mortality. In poor conditions, growth can be zero. However, growth will resume in the model as soon as conditions improve, regardless of the length of time poor conditions persist. To address this limitation, the economic analysis described below includes a case investigating the influence of pasture persistence.

#### 4.1.1. Site Characteristics

Pasture production from paddocks (1 ha) at two locations was modelled to capture the differences in pasture DM responses in regions of southeast Australian with longer and shorter growing seasons. The long-growing season site was based on data from Hamilton, Victoria, which has had a historic average rainfall of 650 mm while the short-growing season site was based on data from Dookie, Victoria, with an historic average rainfall of 550 mm. Rainfall and thus pasture production at Hamilton typically continues later into spring than at Dookie [14]. These sites cover the range of rainfall where a switch from a shallower-rooted species like perennial ryegrass to a deeper-rooted species such as tall fescue or Phalaris could be appropriate.

Soil data, including hydrologic parameters, came from research scenarios developed previously for these areas [28]. A chromosol soil with 25% clay and the default parameters for a high organic matter (2.97%) soil was modelled at the long growing season site. The same chromosol with medium organic matter (2.17%) was modelled at the short growing season site. This allowed for consistent simulations with carbon levels stabilised to long-term rainfall. Chromosols are patchily distributed throughout Victoria and are a common soil type in several pasture regions of the state.

In the model, 25 mature wethers/ha were grazed at the long growing season site and 15 mature wethers/ha were grazed at the short growing season site. The model represents stock as dry sheep equivalents. For the purposes of this study, these values are intended to reflect a level of stocking intensity appropriate to these areas, not a specific farming system. Stocks were removed when dry matter was <0.8 tons DM per hectare and were replaced when dry matter recovered to 1.3 tonnes per ha. Nitrogen fertiliser was applied at both sites at levels that ensure nitrogen did not limit modelled plant growth.

#### 4.1.2. Pasture Species

To quantify the influence of deep roots, three grass species were modelled. The shallower-rooted species was described in the SGS model primarily using default parameters for perennial ryegrass. The intermediate and deep-rooted species differed only in rooting depth and root distribution. The default parameters for perennial ryegrass were validated [24] and include optimal leaf nitrogen composition of 4% and optimal temperature for photosynthesis of 23 °C. Starting at 30 °C growth in the model is impacted by heat, with full heat stress occurring at 35 °C. For all species, the recovery after heat stress (T sum for recovery) was changed from the default value of 100 to 20 based on the findings of Perera et al. [29], who demonstrated the lag between heat stress and recovery was too long using the default parameters. Rooting depths were altered from the perennial ryegrass default values. The root distribution of the shallow-rooting species was similar to that reported for perennial ryegrass with a maximum root depth of 90 cm and 50% of roots occurring in the top 15 cm [30]. The intermediate rooted species has parameters similar to tall fescue with 50% of roots occurring in the top 25 cm and a maximum root depth of 120 cm [30]. The deeper-rooted species had a maximum root depth of 120 cm, but had 50% of roots occurring in the top 40 cm.

#### 4.1.3. Climate Scenarios

In addition to the historic climate, which served as a reference, traditional climate change scenarios (referred to as Future) and scenarios with increased climate variability (referred to as Extremes) were used to investigate the performance of the three species, leading to a total of 18 scenarios. The historic climate was based on data for each site from the Australian Bureau of Meteorology, sourced through the SILO patch point dataset [31] between 1976 to 2013 and this period served as the baseline years for the all climate change scenarios. The traditional climate change scenarios were developed using change factors from an RCP 8.5 scenario for 2080. The ensemble means for rainfall, minimum, and maximum temperature used were the CMIP 5 models available at the Climate Change in Australia website, with the five worst performing models for southern Australia excluded [32]. In this climate model evaluation for five Australian regions only five models had skill scores less than 400 for the southern Australian region. Those were BNU-ESM, CESM1-WACCM, CMCC-CESM, GFDL-ESM2M, IPSL-CM5A-LR. The 35 models included in the ensemble projections include a wide range of variation in projected temperature increases and changes in rainfall [32]. The Extreme climate change scenarios aimed to increase variability of temperature, while preserving the maximum mean temperature, based on the methodology of Harrison [27]. This was done by upscaling all daily maximum temperatures above the median monthly maximum and upscaling only 50% of the values below the median monthly maximum temperature. This resulted in a more variable climate projection and greater frequency of extreme heat. This was done to investigate the impacts of greater variability in temperature on plant responses. All climate change projections included a CO_2_ concentration of 758 ppm based on concentrations for 8.5 RCP in 2080 [33].

Rainfall, temperature, heat stress days, soil moisture availability, and pasture productivity for the scenarios are reported in the results. The biophysical model’s outputs for pasture productivity, pasture ME, and nitrogen application were used in the economic analysis described below. The differences in soil moisture availability, root and pasture growth, and energy intake (MJ) by livestock between the three species were compared across the three climate projections and two sites.

### 4.2. Economic Analysis

For the economic analysis, the scenario used was the re-establishment of a 100-ha paddock. Improved pasture plants were assumed to make up a small proportion of the paddock, the soil P content was low, lime had not been applied in over 15 years, and low energy value, low digestibility grasses and weeds were predominant. To improve the pasture, existing plants were removed, capital fertiliser and lime applied, and an improved pasture species sown into a prepared seedbed. The choice of pasture to sow was one of three species of improved pasture plant described earlier. The only difference in the characteristics of these three plant species was the depth and distribution of plant roots, defined as shallow, intermediate, and deep, as described in Section 4.1.2.

The economic analysis addresses these research questions:

- What is the most likely real return on capital invested, per annum, if a farmer invests to increase the dry matter by sowing one of the three described pasture species?
- Which of the three pasture species would be most profitable to sow considering risk?
- If the intermediate and deep root pasture species has increased persistence compared with the shallow rooted species would this change the conclusion about which pasture species is the best investment to sow in the paddock?

To answer these questions a discounted net cash flow budget of annual net returns over the life of the pasture was used. The numbers in the budget were in real dollars (no inflation) and real opportunity cost of farmer capital. The Modified Internal Rate of Return (MIRR) which measures the return on investment over the life of the project was calculated. The investment was analysed over 30 years which involved several cycles of pasture life.

The key assumptions behind the economic analysis were:

- The results from the biophysical modelling were inputs into the economic analysis. The estimates of growth of pasture dry matter from the biophysical modelling were scaled to 100 ha to represent the consequences of a decision to renovate 100 ha of pasture in a grazing system.
- The two-price method for valuing pasture was used to value the pasture used on farm each month (described below, Section 4.2.3).
- The initial capital investment in establishing the improved pastures was $580/ha.
- The cost of re-establishing the improved pastures during the repeated life cycle was $491/ha. It was assumed there was some of the initial ‘bank’ of capital fertiliser from the start of the project because of annual maintenance applications of fertiliser.
- The investment in pasture improvement was analysed for three climate scenarios. These were historic climate scenario, Future climate scenario and Extremes climate scenario, as explained above (Section 4.1.3).
- Probability distributions were used to:
  ○ Determine the starting year from the 40 years of SGS modelled data.
  ○ Account for a range in the value of pasture.
  ○ Encapsulate the likelihood of establishment failure.

#### 4.2.1. Establishment Assumptions

It was assumed the pasture would each take three years to establish and reach peak annual production (Table 4). A discrete distribution was used to allow for establishment failure, with a probability that this would occur one year in ten. If the pasture did not establish, it was assumed that there would be an additional cost in the subsequent year of $222 per hectare to re-establish it.

#### 4.2.2. Persistence Assumptions

‘Persistence’ was defined in this study as the continuing production of the pasture species at a level of annual production that warrants continuing using that pasture, for a defined time following pasture establishment. The persistence of a pasture over time is determined by factors like insect damage, weeds, grazing management, summer rainfall and temperature, and soil related issues such as compaction, drainage, soil structure, fertility, and pH [34].

**Figure 5:**
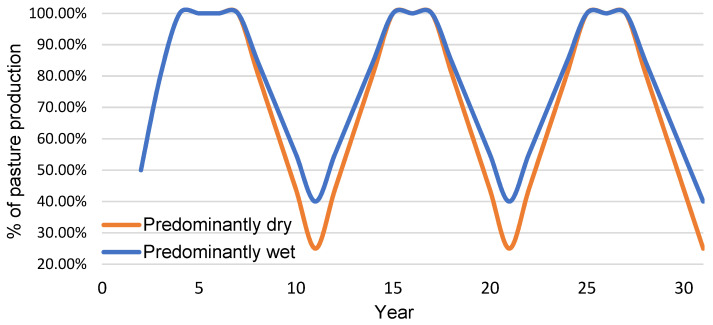
The assumed percentage of modelled pasture growth, attributed to capital investment in pasture improvement, reflecting a 10-year persistence period for both pasture species for a predominately dry 30-year period and a predominately wet 30-year period.

For this study, the persistence of the pasture after ten years was adjusted based on rainfall. If the average annual rainfall was below average 50% or more of the time, the 10-year period was defined as being a predominately dry period. It was assumed that after a predominantly dry period 25% of the pasture remained. If annual rainfall was above average for more than 50% of this period, it was assumed the amount of pasture remaining after the life of the investment was 40%. Figure 5 shows the adjustment to the pasture remaining at year ten to allow for persistence of pasture. It was assumed the farmer managed the other factors that affected persistence.

A second scenario was considered to assess the consequences of the intermediate and deeper-rooted pasture species having increased persistence. According to Nie et al. [35], the persistence of a more shallow-rooted pasture (such as perennial ryegrass) and a deeper-rooted pasture (such as tall fescue) differ. In this case, it was assumed that:

- A pasture sown with the shallower-rooted species would have declined in annual production such that it would need to be renovated after ten years; and
- A pasture sown with a deeper-rooted species, would have declined in annual production such that it would need to be renovated after 15 years.

**Figure 6:**
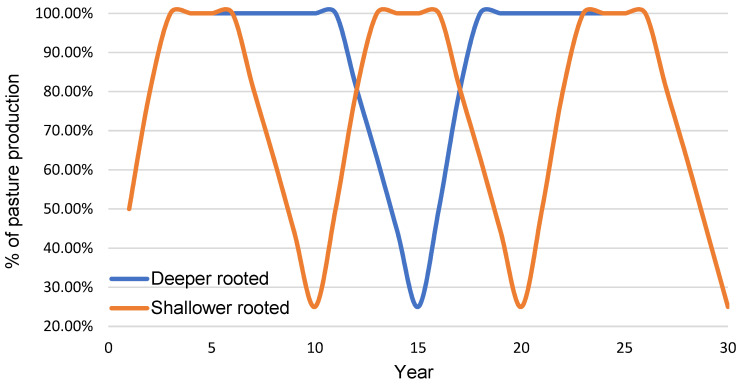
The percentage of modelled pasture growth for the shallower- and deeper-rooted pasture species (to account for establishment differences and differences in the persistence of the different pasture species) based on a predominately dry 30-year period.

Figure 6 displays the percentage adjustment made to the two pasture species during a predominately dry period to reflect the improved persistence of the deeper-rooted pasture.

#### 4.2.3. Valuing Pasture

The method of Hardin and Johnson [36] was used to value the pasture dry matter consumed by livestock. The competitive market price of ME in an economy is the same wherever it is used. The price of ME bought and sold in markets is a good guide to the value of ME in pasture that is used on farms, whether home-grown or supplied from outside the farm. In this study the value of pasture is based on the value of ME supplied by pasture and consumed by livestock as calculated by the biophysical model, which incorporates livestock utilisation and stocking policy.

The value of the pasture that is grown and used on the farm at different times of the year depends on supply of pasture (or ME) and the demand for pasture (or ME) through the year [36].

- At the time in the year when the farm business has low pasture dry matter available (there is more demand for pasture than is supplied) extra pasture grown and used is highly valued. The value of pasture used at this time is equal to the replacement value of ME bought from alternative sources and used during a time of feed shortage (such as grain), or the value of ME conserved and stored in a time of surplus and used later. Note, this ME produced and used on farm cannot be more than the market value of alternative sources of ME off the farm, otherwise it would go to the higher value alternative uses off the farm.
- At the time of the year when there is more pasture supplied than required by animal feed demand, the value of the extra pasture, with further investment, becomes a conserved product to be used later, or the value as agistment at the time it is grown. This value is called the salvage value of the extra ME. Note: the extra ME must be worth more than this value if it is used on farm in the production period.

Thus, there is an upper and lower value of extra pasture produced and used in a farm system in a production period. Surplus pasture has a value equivalent to agistment or standing hay value while ME from pasture dry matter that is in short supply is valued at the value of substitute sources of ME. The true value of the extra pasture used in a farm system lies between the upper (replacement ME) and lower (surplus ME) values.

In this study, as in the method discussed above, the replacement price was used to value the extra pasture grown and used during times when the supply of feed is short relative to the demand for feed by animals. For the regions of interest, this time was defined as being from January to August. The salvage value was used to value the extra pasture grown when the feed supply is surplus to animal demand, which was defined as the period between September and December. This involved modelling pasture growth under historic climate conditions.

A second scenario was considered to account for the impact a changing climate would have on pasture growth and the associated value of pasture over the year. Cullen et al. [37] argued that with a changing climate there will be a change in the seasonal pattern of pasture growth, with a contraction of the spring growing season and higher pasture growth rates in winter and early spring. Perera et al [14] also found that pasture growth patterns have changed over the past 56 years in southeast Australia. To account for such a change in seasonal growth rates occurring at the times when pasture supply is in surplus and in shortage, the extra pasture grown in October to June was valued at the replacement cost and the extra pasture grown in July to September was valued at the salvage value.

The replacement value of ME, such as the price of ME in grain, cannot be defined by a single price for the life of the investment. Equally, the salvage value of surplus ME, which could be the price of standing hay, cannot be defined by a single price for the life of the investment. A probability distribution was used to define the range of possible values the pasture could take over the life of the investment (Table 5).

## 5. Conclusions

Deeper-rooted pasture grasses are often cited as an adaptation to warmer and drier climate in temperate regions. However, this research has shown that pasture production from deep-rooted species is not always substantially greater. This study in southern Australia demonstrated that deeper-rooted species could be a good adaptation response, assisting in maintaining farm profitability, for farmers in higher rainfall areas where soil moisture was available for the deep-rooted species to access. However, soil moisture limited the adaptation benefit for farmers in the lower rainfall areas. These results highlight that the development of climate change adaptation options needs to consider local conditions, including climate and soil types, to avoid maladaptive outcomes. Biophysical farm systems models are an important tool to achieve this. This research has also shown that pasture persistence is important to the profit of climate adaptation responses. Persistence pays and should be incorporated when modelling climate impacts. Management options that focus on summer dormancy and improve pasture persistence during hot, dry periods would likely provide more benefit for dry areas than a focus on deep-rooted species, and the potential for such options in a future climate warrants further investigation.

## Figures and Tables

**Table 1:** Climate characteristics of the growing (April–October) and non-growing (November–March) seasons on the longer and shorter growing season sites under three climate scenarios (standard deviation in parentheses).

Scenarios	Climate	Season	Average Maximum Temperature (°C)	Average Minimum Temperature (°C)	Average Rainfall (mm)
Longer growing season site	Historic	Growing	15.5 (3.9)	6.2 (3.2)	451 (79)
		Summer	24.1 (6.0)	10.4 (3.7)	190 (52)
	Future	Growing	18.4 (4.5)	9.0 (4.5)	430 (74)
		Summer	27.3 (6.6)	13.5 (4.8)	173 (54)
	Extremes	Growing	18.4 (5.3)	7.3 (3.9)	430 (74)
		Summer	27.3 (7.6)	11.7 (4.4)	173 (54)
Shorter growing season site	Historic	Growing	15.5 (4.6)	4.5 (3.8)	351 (119)
		Summer	26.9 (5.3)	12.0 (4.2)	197 (93)
	Future	Growing	18.6 (5.2)	6.9 (5.1)	316 (106)
		Summer	30.4 (5.8)	15.0 (5.1)	198 (97)
	Extremes	Growing	18.6 (6.0)	5.4 (4.6)	316 (106)
		Summer	30.4 (6.9)	13.6 (4.9)	198 (97)

**Table 2:** The monthly average number of heat stress days (max >35 °C or average >23 °C) per year at each site.

Season	Longer-Growing Season			Shorter-Growing Season		
	Historic	Future	Extremes	Historic	Future	Extremes
Dec	5.2	9.7	10.1	9.2	17.7	16.7
Jan	8.3	13.0	13.6	15.7	22.7	19.9
Feb	8.6	13.4	13.3	14.2	20.9	17.8
March	0.8	9.7	10.1	6.1	13.7	15.2
April	0	3.8	4.3	0.2	3.0	4.1
May	0	0.3	0.7	0	0.1	0.1
June	0	0	0.2	0	0	0
July	0	0	0.2	0	0	0
August	0	0.1	0.2	0	0	0.1
September	0	0.4	0.8	0	0.6	1.0
October	0.5	2.2	3.4	0.7	3.6	4.7
November	2.6	6.3	7.0	4.3	17.7	12.7
Annual	31.3	58.9	64.0	50.5	93.7	92.3

**Table 3:** For each scenario, spring (September–November) and annual pasture growth (t DM/ha) and annual intake in GJ of ME are displayed. Values in parentheses are standard deviations.

Site	Climate	Variable	Deep-Rooted Species	Intermediate-Rooted Species	Shallow-Rooted Species
Longer growing season site	Historic	Spring growth	3.7 (0.9)	2.7 (0.8)	2.4 (0.7)
		Annual growth	6.5 (1.1)	6.1 (1.1)	6.0 (1.1)
		Annual intake	48.3 (9.8)	46. 7 (9.7)	44.9 (9.6)
	Future	Spring growth	3.2 (0.8)	2.2 (0.7)	1.9 (0.6)
		Annual growth	6.7 (1.1)	6.3 (1.1)	6.3 (1.1)
		Annual intake	44.0 (9.5)	42.6 (9.3)	41.3 (9.3)
	Extremes	Spring growth	3.0 (0.8)	2.1 (0.7)	1.8 (0.6)
		Annual growth	6.7 (1.2)	6.4 (1.1)	6.4 (1.1)
		Annual intake	44.5 (9.5)	42.9 (9.2)	41.6 (9.0)
Shorter growing season site	Historic	Spring growth	2.5 (0.8)	1.9 (0.8)	1.6 (0.7)
		Annual growth	5.0 (2.0)	4.9 (1.8)	4.8 (1.7)
		Annual intake	27.0 (9.6)	25.5 (10.1)	23.2 (9.8)
	Future	Spring growth	1.6 (1.0)	1.2 (0.9)	1.1 (0.8)
		Annual growth	5.0 (2.2)	5.1 (2.1)	5.2 (1.9)
		Annual intake	24.8 (8.1)	22.9 (9.2)	20.3 (9.7)
	Extremes	Spring growth	1.5 (1.0)	1.2 (0.9)	1.1 (0.8)
		Annual growth	4.9 (2.2)	5.1 (2.1)	5.2 (1.9)
		Annual intake	24.8 (8.3)	23.0 (9.6)	20.6 (10.0)

**Table 4:** The assumed percentage of pasture grown during establishment until peak production.

Year after Establishment	1st	2nd	3rd	4th
Potential growth (%)	50%	80%	100%	100%

**Table 5:** The probability distribution and range of values considered for the value of pasture at different times in the year in $/t and in $/MJ ME.

Species	Distribution	P5	P50	P95
Upper value of pasture (replacement cost)	PertAlt	$190/t $0.017/MJ ME	$260/t $0.023/MJ ME	$350/t $0.031/MJ ME
Lower value of pasture (salvage cost)	PertAlt	$90/t $0.013/MJ ME	$130/t $0.018/MJ ME	$190/t $0.026/MJ ME

^1^ The upper value of pasture was assumed to have 12.5 MJME/kg DM whereas the lower value of pasture was assumed to have 8 MJME/kg DM. It was assumed 90% DM for both upper and lower value pasture. The lower value of pasture was based on market prices for hay less than $60/t DM to estimate a standing hay price. To value the ME of pasture the following formula was used: ME_$_ = DM_$_/ME_DM_, where ME_$_ is the value of 1 MJ ME pasture.
